# The Bodies and Minds of Children

**Published:** 1891-09-05

**Authors:** 


### The Doctor's Armchair.

### THE BODIES AND MINDS OF CHILDREN.

The International Congress of Hygiene and Demography has fully recognised its duties to the world of childhood. All grown-up people owe much to children, and all who are well-conditioned are anxious to discharge their obligations. The schoolmaster and the doctor have at length shaken hands, and they are now disposed to work together without jealousy for the greatest advantage of the children under their charge.

Most readers have a general idea of the way in which the schoolmaster and the schoolmistress deal with boys and girls. But very few know how the doctor conducts his work in school. A few facts brought before the International Congress by Dr. Francis Warner will illuminate the subject. Dr. Warner has conducted certain investigations for the British Medical Association, and he has, therefore, had the advantage of dealing with very large numbers of children. He made special observations in great detail on fifty thousand boys and girls attending the schools of the London School Board.

Dr. Warner's general method is this: In a large well-lighted hall he has the children intended for observation drawn up in ranks, a standard at a time, or in groups of about forty, so that the observer can see each individual clearly. It is desirable to fix a child's eyes whilst he is under observation, and in order to do this Dr. Warner holds up a pencil with a shilling balanced on its end. All eyes being fixed on the balanced shilling, the trained observer, says the doctor, can read off the physiognomy of the individual features and their parts, the facial action and expression, the eye movements, the balance of the head and body, as quickly as a printed line. The children are then requested to hold out their hands straight; and the action and balance are noted as a further indication of the condition of the nerve system. Finally, the palate of each child is inspected. At the successive stages of the investigation those children who present deviations from the normal standard of form and action are asked to stand aside; and any cases of abnormality which the Medical Inspector may have passed over are then indicated by the teacher, who, from his daily contact with the children, has had ample opportunities of finding out any who have peculiarities that are not obvious. It is clear that by a method of this kind careful and intelligent investigators may easily single out every boy and girl in every group who has any sort of abnormality of mind or body. Persons who are disposed to think that it is carrying things a little too far to make all this fuss about children, will do well to note the number of abnormal cases in the fifty thousand examined by Dr. Warner. There were nearly ten thousand who had one or more peculiarities either of body or mind. And those pecularities were not mere trifles; they were disabilities, and for the most part important ones. Here, for example, are cases which illustrate the kind of disabilities: Of the 10,000 abnormal children more than 3,000 presented defects in bodily development or indications of nerve abnormality. Nearly 800 were not only defective in development but showed abnormal nerve signs and evidences of low nutrition as well. No fewer than 67 of the 10,000 were deaf or partially deaf, whilst 341 were crippled, deformed, or maimed. As many as 54 were the subjects of epileptic fits.

Now the object of Dr. Warner and of his colleagues at the International Congress was not to excite pity; nor is that our purpose. Science has put us in possession of these facts about Board School children; but what is true of Board School children is true, though in differing measure, of all other children. Parents of the upper and middle classes may take it for granted that if fifty thousand of their children were examined by Dr. Warner's method a great many would be found to present the defects, abnormalities, and disabilities which were discovered in the fifty thousand Board School children. Many would be cripples, many deaf; many would have arched palates, some would be epileptic, and a large number would be of low nutrition, and otherwise unequal to their fellows of the same age.

The discussion which followed Dr. Warner's address revealed facts with which many intelligent persons are more or less acquainted, but which are by no means universally taken into account by parents and school teachers. It seems certain that, taking the whole population of this country, about one child in every five or six is feebler in mind and body than the average. He cannot do the school work of the average child: he will never be able to do the world's work of the average man. But by the prevailing methods of school life, home life, and world life he is put to exactly the same tasks as the average boy or man, and he is expected to do them.

What is the result of this unintelligent method of procedure? It is twofold: great cruelty is inflicted upon the unhappy child or man who falls below the average mental and bodily level. That is the first result. But there is a second, which inflicts just punishment upon the blundering stupidity of those countries that fail to distinguish the abnormal and the unhappy from the strong and vigorous in body and mind. The 10,000 defectives in the 50,000 examined will, in all probability, largely help to fill our prisons and our county lunatic asylums, and will thus have their revenge at least on the pockets of their more fortunate but stupid contemporaries. Dr. Shuttleworth, quoting from one of Dr. Waring's works, affirmed that "the excitable boy, always at home with a headache, might grow up without education, and so excitable that in a moment of temper he might do something which would bring him within reach of the law." He also mentioned the case of a little girl who was always in trouble at school for stealing. Dr. Waring found that she was suffering from chorea and heart disease. When she was sent to a quieter school she got better and left off stealing. Dr. Fletcher Beach stated that the Charity Organisation Society had found that many of the under-average children of our schools drifted finally to the workhouses and into the criminal classes; and Dr. Langdon Down attested that many years ago, when he investigated closely the physical and mental condition of the inhabitants of one of Her Majesty's prisons, he was struck by the fact that a large number of the so-called criminals were really feeble-minded people.

Parents and guardians, as well as County Councillors and other public men, must take to heart the facts of science here set forth. You cannot put the round child into the square hole, any more than you can put the round man. Science and plain sense are entirely at one here. Parents must study their own children, and get the intelligent doctor to help them. County Councillors and school managers and teachers must also study the children under their charge, and must seek the guidance of the man whose lights are physiological and scientific. If by our stupid management of defective children we force them into the pathways of criminality and lunacy we are worse criminals and greater lunatics than they are. Give them a chance. Separate the normal from the abnormal, and let each have tasks suited to his capacity. We must not be jealous of science, nor must we treat her with contempt. Her commands, like all commands that commend themselves by their sweet reasonableness, are to be promptly and cheerfully obeyed.

